# A Study of the Effect of Temperature on the Capacitance Characteristics of a Metal-*μ*hemisphere Resonant Gyroscope

**DOI:** 10.3390/s24217088

**Published:** 2024-11-04

**Authors:** Xiangxian Yao, Hui Zhao, Zhong Su, Xibing Gu, Sirui Chu

**Affiliations:** 1 Beijing Key Laboratory of High Dynamic Navigation Technology, Beijing Information Science and Technology University, Beijing 100192, China; 2023020388@bistu.edu.cn (X.Y.); zhhui@bistu.edu.cn (H.Z.); 2023020390@bistu.edu.cn (X.G.); 2023020380@bistu.edu.cn (S.C.); 2 Key Laboratory of Modern Measurement and Control Technology, Ministry of Education, Beijing Information Science and Technology University, Beijing 100192, China

**Keywords:** M-*μ*HRG, temperature effect, static capacitance, dynamic capacitance, nonlinear capacitance variation

## Abstract

Metal-μhemispherical resonant gyros (M-μHRGs) are widely used in highly dynamic navigation systems in extreme environments due to their high accuracy and structural stability. However, the effect of temperature variations on the capacitance characteristics of M-μHRGs has not been fully investigated, which is crucial for optimizing the performance of the gyro. This study aims to systematically analyze the effect of temperature on the static and dynamic capacitances of M-μHRGs. In this study, an M-μHRG structure based on a 16-tooth metal oscillator is designed, and conducted simulation experiments using non-contact capacitance measurement method and COMSOL Multiphysics 6.2 finite element simulation software in the temperature range of 233.15 K to 343.15 K. The modeling analysis of the static capacitance takes into account the thermal expansion effect, and the results show that static capacitance remains stable across the measured temperature range, with minimal effect from temperature. The dynamic capacitance exhibits significant nonlinear variations under different temperature conditions, especially in the two end temperature intervals (below 273.15 K and above 313.15 K), where the capacitance values show local extremes and fluctuations. In order to capture this nonlinear behavior, the experimental data were smoothed and fitted using the LOESS method, revealing a complex trend of the capacitance variation with temperature. The results show that the M-μHRG has good capacitance stability in the mid-temperature range, but its dynamic performance is significantly affected at extreme temperatures. This study provides a theoretical reference for the optimal design of M-μHRGs in high- and low-temperature environments.

## 1. Introduction

Axisymmetric-shell resonant gyroscopes are divided into two types of excitation: one involves applying the excitation force to a fixed point in the circumferential direction of the shell, i.e., contact axisymmetric-shell resonant gyroscope; the other involves equivalently applying the excitation force to the wave amplitude of the vibration pattern, and the energy supplement is in tune with the intrinsic vibration of the resonator, which puts the oscillator in an equivalent free resonant state, i.e., non-contact axisymmetric-shell resonant gyroscope. Among the latter, a typical construction is the hemispherical resonant gyro (HRG), which is a solid fluctuating gyro based on the Götzsch effect. It utilizes the Coriolis effect of standing waves during the vibration of an axisymmetric shell to measure the angular velocity of a rotating body [1,2,3]. An HRG has the comprehensive advantages of high accuracy, high reliability, simple structure, small size, and lightweight nature, and it has broad application prospects in the field of deep space. The University of Michigan and the University of California, among others, have developed mature HRG products [4,5,6]. However, with the advancement of technology and the improvement of application requirements, metal resonant gyroscopes are better able to cope with high overloads and extreme environments due to their higher structural strength and environmental adaptability [7]. In recent years, the team of Raspopov V. Ya. has proposed a new hemispherical resonant gyro design using a metal resonator, which significantly improves the stability and reliability of the device in harsh environments [8]. The team at the Beijing University of Information Science and Technology designed a metal micro-hemispherical resonator with rectangular teeth and proposed a vibrator design and optimization method for the metal micro-hemispherical resonant gyroscope [9], as well as optimizing the structure of the composite electrode-driven metal-shell resonant gyroscope and the excitation method [10].

The capacitance characteristics determine the performance index of the metal resonator gyroscope. Traditionally, metal-shell resonator gyroscopes use a contact method for signal acquisition and processing. This requires the connection of eight piezoelectric electrodes, which are distributed along the drive and detection axes of the oscillator [11,12]. However, the use of piezoelectric control forces for contact excitation and detection can affect the characteristics of the vibrator. In particular, under a wide range of angular motion inputs, a standing wave deficit may be observed [13]. In order to minimize the errors of the contact method, non-contact discrete plate electrodes and three-dimensional structures are often used in the operation of metal-shell resonator gyroscopes. This approach eliminates the need for contact excitation and detection, resulting in a more streamlined gyroscope [14,15] construction process. This non-contact arrangement allows a planar capacitive structure to be formed between the oscillator and electrodes, which is more accurate in capacitance value measurements and makes it easier to calculate capacitance values compared to non-planar capacitive structures.

Zhang et al. designed a weak capacitance detection circuit for detecting the capacitance generated by a hemispherical resonant gyroscope, which shows high sensitivity in detecting very small capacitance changes. However, this circuit is mainly designed for capacitance changes under static or quasi-static conditions, and it is difficult to accurately capture the characteristics of capacitance changes in high-speed dynamic environments. In addition, the anti-interference ability of the detection circuit still needs to be strengthened, especially if the stability of the high-frequency environment is deficient [16]. The real-time calibration method for capacitance displacement detection proposed by Sun Jiangkun et al. cleverly utilizes the relationship between the first-order and third-order harmonic components of the detection signal for calibration. Although this innovative method improves calibration accuracy, its adaptability to complex vibration modes and high harmonics is poor. Especially in the face of complex situations where multiple harmonics coexist, the calibration effect may be limited, leading to an increase in errors [17]. By studying the uniformity of HRG assembly capacitance, Yu Hui et al. proposed a fast uniformity-based attitude error identification algorithm and used a support vector regression (SVR) model to construct a nonlinear relationship between the capacitance and attitude error. Although the algorithm showed high accuracy in assembly error identification, it is only optimized for specific types of assembly errors, and there is still inadequate identification of capacitance inhomogeneity and more complex error patterns under dynamic working conditions. Additionally, the real-time performance of the algorithm still needs to be improved [18]. Yuan Lishan et al. proposed a high-precision mounting and welding method for a flat-electrode hemispherical resonant gyro. Although there was a significant improvement in ensuring assembly accuracy, the detection capacitance is still limited to 1∼5 pF, which makes it difficult to cope with a wider range of capacitance variations [19]. Yang et al. conducted an in-depth exploration of the thermal equilibrium characteristics of HRGs, demonstrating that even minor temperature variations can significantly affect their dynamic stability [20]. Despite these advancements, the specific impact of temperature on the capacitance characteristics of HRGs remains largely unexplored in current literature. This represents a critical gap, particularly considering the pivotal role of capacitance in maintaining precise operational stability in dynamic environments.

The aim of this study is to systematically analyze the effect of temperature on the static and dynamic capacitance characteristics of M-μHRGs. The trends of capacitance and its nonlinear characteristics in the low-, medium-, and high-temperature intervals are found by conducting experiments on an M-μHRG under different temperature conditions. The study of static capacitance addresses its stability under temperature variation, while the study of dynamic capacitance focuses on the complex response to time and temperature. In this paper, the temperature dependence of dynamic capacitance is explored in detail through experimental data analysis and the LOESS fitting method, revealing the capacitance fluctuation phenomenon in different temperature intervals.

## 2. Capacitor Structure Design

### 2.1. Oscillator Structure

**Figure 1:**
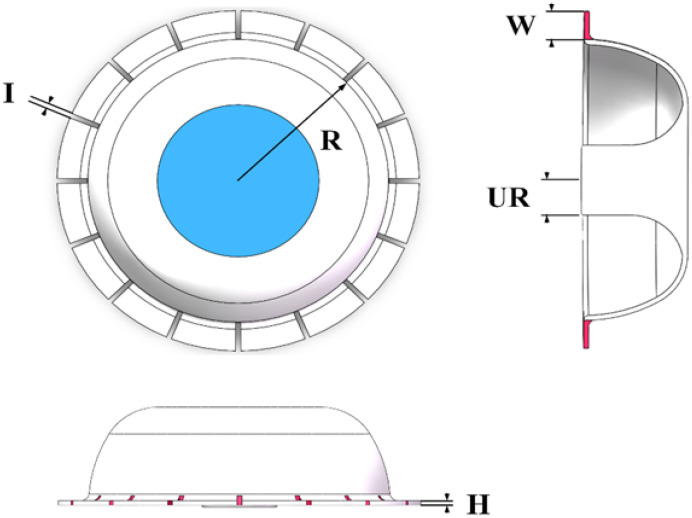
Schematic diagram of the metal micro-hemispherical oscillator.

As shown in Figure 1 and Table 1, a 16-tooth vibrator was selected for this study, and the selected material was steel 3J33B with a high yield strength and high overload capacity, Young’s modulus of 200,000 MPa, material density ρ of 7850 kg/m^3^, Poisson’s ratio μ of 0.3, coefficient of thermal expansion of 3.5 $\times \;10^{-6}$/K, compressive yield strength of 250 MPa, and tensile yield strength of 250 MPa.

### 2.2. Capacitor Construction

**Figure 2:**
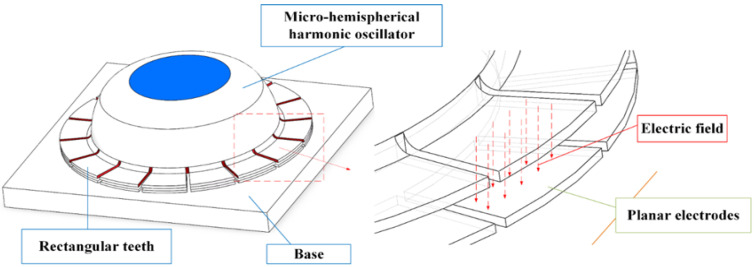
Schematic diagram of M-μHRG capacitance.

Sixteen electrodes [21], each covering an angle of 22.5°, are uniformly distributed around the circumference of the vibrator, of which eight electrodes are used for the excitation and signal detection of the vibrator, while the other eight electrodes are used for compensation correction. The structural properties of the HRG dictate that its performance is hardly affected by the concentric error between the resonator and the electrode base induced by the assembly process. On the contrary, the concentric error can seriously affect the performance of the classical 3D-electrode HRG. Therefore, this is a major advantage of planar-electrode HRGs [22]. By applying an AC voltage between the oscillator and the electrode to excite the oscillator and generate resonance, the amount of change in the capacitance of the flat capacitor between the oscillator and the electrode is detected. The detected change in capacitance is converted to a change in voltage on the detected planar electrode, which sensitizes the change in the oscillator’s vibration pattern. Between the bottom surface of the oscillator and the electrode, some small capacitance will form; these capacitances can be approximated as a flat capacitor, as shown in Figure 2.

## 3. M-μHRG Capacitance Modeling

In this section, the static and dynamic capacitances are modeled and analyzed separately. For static capacitance, the capacitance model was first established under ideal conditions, and the capacitance value was mainly calculated based on the distance between the electrode and the oscillator, the overlap area, and the material’s dielectric constant. Then, the static capacitance model under the influence of temperature was constructed by introducing the thermal expansion effect of the material and correcting for the change in the geometry of the model due to temperature changes in practical applications. Next, for the modeling of dynamic capacitance, based on the consideration of the vibrational displacement of the vibrator, the change in the distance between the electrode and the vibrator with time was analyzed. The time-varying model of dynamic capacitance was established and further combined with the temperature change and other factors, allowing the effect of temperature on dynamic capacitance to be deduced.

### 3.1. Static Capacitance Modeling

#### 3.1.1. Static Capacitance Modeling in the Ideal Case

Static capacitance refers to the capacitance of electrodes and oscillators under static conditions, which is generated by the electric field under fixed geometry and material properties. For the M-μHRG, the capacitance mainly depends on the distance between the oscillator and the electrodes, the overlap area, and the dielectric constant of the material. Under ideal conditions, the edge effect of the capacitance can be neglected [23], according to the parallel plate capacitance equation

(1) $$C=\frac{\varepsilon S}{d_{0}}$$

where ε is the vacuum dielectric constant, *S* is the overlap area between the teeth and the electrodes, and $d_{0}$ is the initial capacitance distance, with a size of 0.05 mm.

#### 3.1.2. Static Capacitance Modeling Under the Influence of Temperature

Assuming that the material undergoes thermal expansion at temperature T, the change in geometry between the oscillator and the electrode can be expressed as

(2) $$d_{T}=d_{0}\left(1+\beta \Delta T\right)$$

where β is the thermal expansion coefficient of the material and ΔT = $T-T_{0}$ is the temperature change relative to the reference temperature. The relative dielectric constant of the material also changes with temperature. In order to analyze the effect of temperature on static capacitance more deeply, based on the temperature dependence of the thermal expansion and dielectric constant of the material, the capacitance model can be expressed as

(3) $$C\left(T\right)=\frac{\varepsilon_{0}\varepsilon_{r}\left(T\right)A\left(T\right)}{d_{0}\left(1+\beta \Delta T\right)}$$

where $\varepsilon_{r}\left(T\right)$ denotes the change in the material’s dielectric constant with temperature and A(T) denotes the change in the overlap area between the electrode and the oscillator due to thermal expansion effects. In most cases, especially in a stationary state, the changes in $\varepsilon_{r}\left(T\right)$ and A(T) relative to the changes in area and spacing can be ignored. Therefore, the whole model can be simplified as

(4) $$C\left(T\right)\approx \frac{\varepsilon_{0}\varepsilon_{r}A_{0}}{d_{0}\left(1+\beta \Delta T\right)}$$

The effect of βΔT is very limited due to the small coefficient of thermal expansion of the material, which means that the effect of temperature change on the electrode spacing is very small. It can be inferred from the model that the static capacitance value does not change significantly with temperature in the regular temperature range.

### 3.2. Dynamic Capacitance Modeling

#### 3.2.1. Dynamic Capacitance Modeling in the Ideal Case

The distance between the rectangular teeth of the oscillator and the planar electrodes varies periodically because the oscillator is in a four-wave belly vibration mode during the operating mode [24].

**Figure 3:**
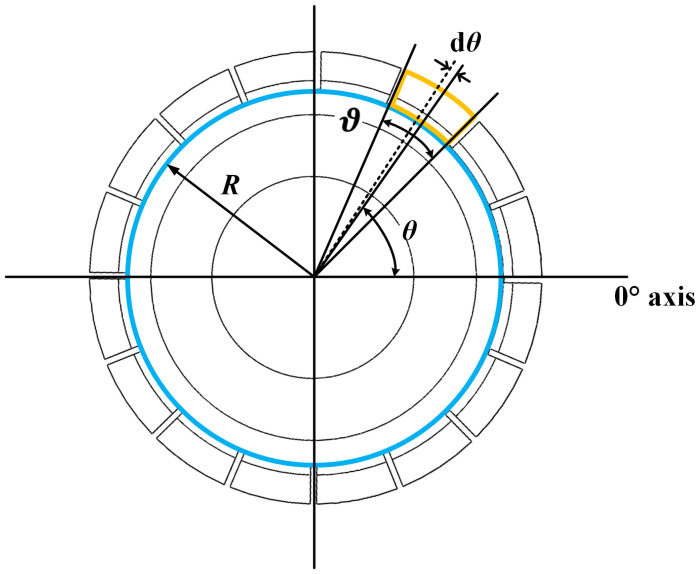
Top view of the M-μHRG capacitor model.

The top view of the M-μHRG capacitance model is shown in Figure 3. θ is the angle between the centerline of a certain electrode and the 0° electrode axis, i.e., the electrode azimuth angle, ϑ is the central angle corresponding to the electrode. When the oscillator vibrates, the distance *d* between the rectangular teeth of the oscillator at the electrode azimuth angle θ and the planar electrode on the base can be expressed as [25,26]

(5) $$d=d_{0}-\left[x\left(t\right)cos2\theta +y\left(t\right)sin2\theta \right]$$

where x(t)cos2θ+y(t)sin2θ is the vibrational displacement of the oscillator at θ. Therefore, the variation of any capacitance $C_{d}$ with time can be expressed as

(6) $$C_{d}=\int_{\theta_{i}-\vartheta /2}^{\theta_{i}+\vartheta /2}\frac{\varepsilon S}{d}d\theta$$

where $\theta_{i}$ is the electrode azimuth of the ith electrode.

#### 3.2.2. Dynamic Capacitance Modeling Under the Influence of Temperature

**Figure 4:**
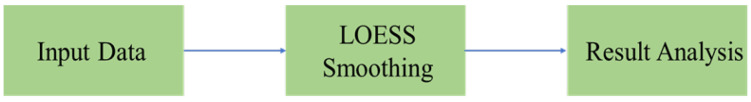
Flowchart of the LOESS fitting method.

As shown in Figure 4, in order to study the effect of temperature on dynamic capacitance, this paper adopts the LOESS (Locally Estimated Scatterplot Smoothing) method, which uses experimental data to fit the nonlinear relationship between temperature and dynamic capacitance. The LOESS method captures the localized characteristics of the data through locally weighted regression, which is more capable of capturing the localized fluctuations and nonlinear trends in the data.

The core of the LOESS method is to perform a locally weighted regression for each temperature point $T_{i}$ to solve for the capacitance-fitted value $C(T_{i})$ at that point. In LOESS, for a given temperature $T_{i}$, the fitted value $C(T_{i})$ is calculated by weighted least squares

(7) $$C\left(T_{i}\right)=\sum_{j=1}^{n}\omega \left(T_{i},T_{j}\right)f\left(T_{j}\right)$$

where $C(T_{i})$ is the fitted value of capacitance at temperature $T_{i}$, $f(T_{j})$ is the original capacitance value corresponding to temperature $T_{i}$, and ω$\left(T_{i},T_{j}\right)$ is the weight function that measures the influence of the distance between temperature $T_{i}$ and $T_{j}$ on the regression results. The expression of the weighting function is

(8) $$\omega \left(T_{i},T_{j}\right)={\left(1-\left(\frac{{\left|T_{i}-T_{j}\right|}^{3}}{d(T_{i})}\right)\right)}^{3}$$

where $d(T_{i})$ denotes the effective distance within the neighborhood of point $T_{i}$. The LOESS method weights the data points according to their distances, with closer data points receiving higher weights and more distant data points receiving lower weights. This weighting function ensures the accuracy of the local fit within the neighborhood of the temperature point $T_{i}$.

In order to cover the typical operating temperature range of metal miniature hemispherical resonant gyroscopes in practical application environments, this study collected 23 sets of average dynamic capacitance values at different temperatures within 233.15 K ≤ T ≤ 343.15 K and smoothed and fitted these data using the LOESS method. Meanwhile, temperature has a significant effect on the thermal expansion and capacitance characteristics of the material, so the present temperature range is sufficient to comprehensively evaluate the performance variation of the resonator at different temperatures.

The choice of the smoothing parameter α plays a key role in the smoothness of the results and the ability to capture local features. In this paper, α = 0.3 is selected. A value that is too small for α will lead to the curve being too close to the original data, which may introduce noise; conversely, a value that is too large for α may make the curve excessively smooth and cause a loss of local capacitance fluctuation information.

The global capacitance–temperature relationship curve is obtained by moving the local window over the entire temperature range and calculating and fitting the capacitance value at each temperature point, point by point.

## 4. Thermo-Mechanical-Electrical Coupling Simulation

**Figure 5:**
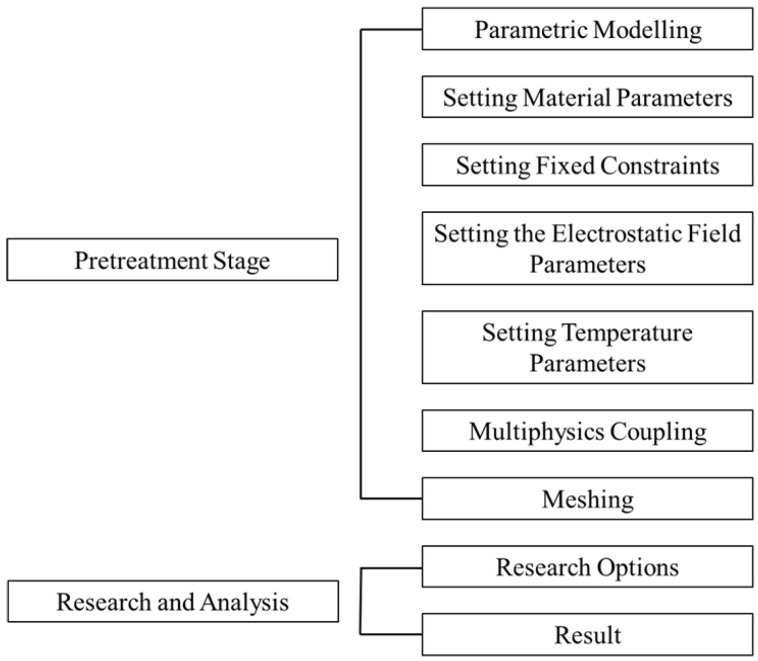
M-μHRG finite-element simulation flow.

For the dynamic capacitance analysis of M-μHRG vibrators under the action of electrostatic excitation electrodes, a multi-physics field coupling method is generally used for finite-element simulation. Through the analytical method of coupled simulation involving solid mechanics, electrostatics, and thermodynamics, AC and DC voltages are applied to the electrostatic electrodes to analyze the dynamic capacitance between the flat-plate electrode and the vibrator in different cases when the vibrator reaches the operating resonant frequency. The specific simulation steps are shown in Figure 5.

### 4.1. M-μHRG Finite-Element Modeling

**Figure 6:**
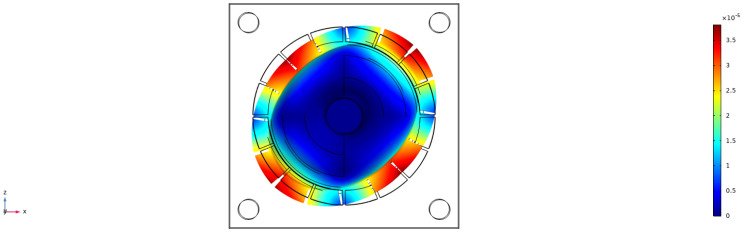
Second-order modal diagram of the oscillator.

In this paper, the oscillator is energized by setting the AC excitation voltage to produce resonant motion. As shown in Figure 6, the oscillator is simulated in the solid mechanics module, and the resonant frequency of the oscillator is obtained as 6850.6 Hz in the second-order mode, with the operating mode being the four-wave belly mode. The variation range of the data and the simulation step size are shown in Table 2.

### 4.2. Static Capacitance Simulation Under the Influence of Temperature

**Figure 7:**
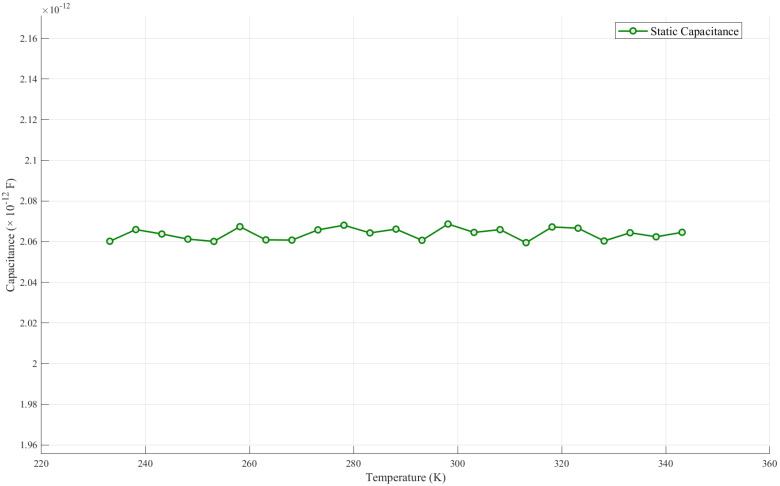
Temperature and static capacitance dependence plot.

Figure 7 shows in detail how the static capacitance varies over a temperature range of 233.15 K to 343.15 K. The static capacitance is also shown in the figure. By observing the data in the figure, it can be seen that the static capacitance value always stays around 2.064 × $10^{-12}$ F. Despite the temperature fluctuations over a wide range, the static capacitance is not significantly affected. This phenomenon indicates that the static capacitance characteristics of the system show a high degree of stability in this temperature range and are able to effectively resist changes in external temperature. It also verifies the correctness of the model.

### 4.3. Dynamic Capacitance Simulation Under the Influence of Temperature

**Figure 8:**
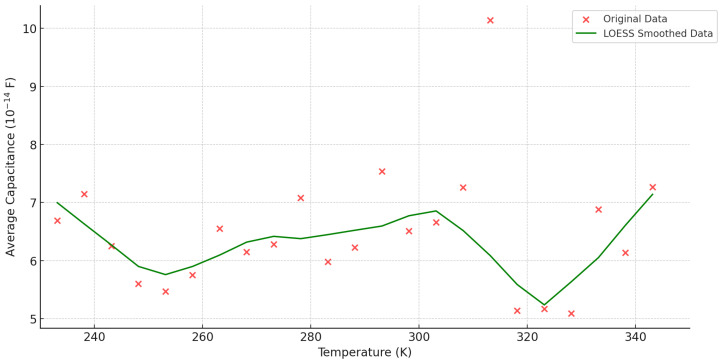
Plot of temperature versus average dynamic capacitance.

In this study, 23 different temperature conditions were set by simulation. Under each temperature condition, the system collected dynamic capacitance values at a frequency of 10 times per second, with a recording time of 20 s, resulting in a total of 4600 sets of data. In order to improve the stability and reliability of the data, the 200 dynamic capacitance data points under each temperature condition were averaged, and finally, 23 average dynamic capacitance values were obtained. Figure 8 demonstrates the relationship between temperature and average dynamic capacitance, and the data were analyzed using LOESS smooth fitting.

As can be seen in Figure 8, the effect of temperature on the average capacitance exhibits significant nonlinear characteristics. The capacitance does not vary monotonically over the entire temperature range but instead exhibits multiple local extremes and fluctuating trends. This complex variation can be better captured by LOESS fitting while avoiding the problems of overfitting or underfitting.

In the low-temperature interval (233.15 K to 253.15 K), the capacitance value decreases from 6.69 × $10^{-14}$ F to a minimum of 5.47 × $10^{-14}$ F, reaching a particularly low value at 253.1 K. The capacitance value of the material is also reduced in the low-temperature interval (233.15 K to 253.15 K). This is because in low-temperature environments, an increase in temperature often leads to a decrease in the dielectric constant. Due to the rapidly changing electric field and polarization response involved in dynamic capacitance, temperature changes can affect the polarization process, dielectric loss, and thermal excitation effects, resulting in a more significant impact of the dielectric constant on capacitance during dynamic changes compared to static states.

As the temperature increases to the mid-temperature range (253.1 K to 313.15 K), the capacitance gradually rises from its lowest point and reaches a local maximum of 10.14 × $10^{-14}$ F at 313.15 K. This is due to the enhancement of the thermal expansion effect of the material, which results in a smoother migration of charge carriers through the material and enhances the capacitive response. In addition, the material structure tends to be thermally stabilized with increasing temperature, which further enhances the capacitive performance.

In the high-temperature range (313.15 K to 343.15 K), the dynamic capacitance of the system exhibits a “peak drop rise” pattern. At 313.15 K, the capacitance reaches its maximum value of 10.14 × $10^{-14}$ F due to the enhancement of the polarization effect, leading to a brief increase in the dielectric constant. Subsequently, the capacitance sharply decreases to a minimum value of 5.09 × $10^{-14}$ F between 318.15 K and 328.15 K due to thermal disturbance disrupting the dipole arrangement and increasing the electrode spacing caused by thermal expansion. Finally, the capacitance gradually increases from 328.15 K and reaches 7.27 × $10^{-14}$ F at 343.15 K, indicating that the dielectric properties tend to stabilize at high temperatures. This is because after the temperature rises to a certain degree, the microstructure inside the material may rearrange, forming a relatively stable state, thereby causing the dielectric constant to stabilize. At this point, although thermal disturbances still exist, their impact is gradually offset by the adaptive structural changes of the material, so the capacitance exhibits stable behavior.

Overall, temperature has a complex and significant effect on the dynamic capacitance of M-μHRGs, especially at extreme temperatures, where the capacitance values exhibit significant fluctuations. The decrease in capacitance in the low-temperature interval indicates that the material’s electrical performance is limited in low-temperature environments, while in the mid-temperature interval, the capacitance exhibits a rebound, suggesting that the material gradually tends to become thermally stabilized. In the high-temperature range, significant fluctuations in capacitance reflect the impact of material parameter changes on capacitance performance at elevated temperatures. This result suggests that the thermal expansion effect of materials and their electrical properties must be taken into account when designing precision sensing devices for high- and low-temperature operating environments to ensure the stability and reliability of the system in extreme conditions.

## 5. Conclusions

In this study, the effect of temperature on the static and dynamic capacitances of M-μHRGs is analyzed in depth. The experimental results show that the static capacitance remains highly stable within conventional temperature intervals and is minimally affected by temperature variations, demonstrating the temperature robustness of M-μHRGs for precision sensing applications. However, the dynamic capacitance exhibits significant nonlinear characteristics at different temperatures, especially in low- and high-temperature intervals, where the dynamic capacitance values show significant fluctuations. This suggests that temperature variations can have a non-negligible impact on the dynamic performance of M-μHRGs. Due to the sensitivity of the dynamic capacitance to temperature variations, future work will focus on a compensation strategy for temperature drift to ensure the performance stability of M-μHRGs at extreme temperatures. The development of effective temperature compensation mechanisms is essential to enhance the device’s application in aerospace and other harsh environments. The results provide a theoretical basis for further optimizing the design of M-μHRGs in the future, especially in terms of improving dynamic capacitance stability and reducing the impact of temperature drift on performance.

## Figures and Tables

**Table 1:** Oscillator structure parameters.

Parameter Name	Basic Value (mm)
Main Body Radius (R)	6.0
Tooth Thickness of Rectangular Teeth (H)	0.2
Tooth Length of Rectangular Teeth (W)	1.2
Spacing of Rectangular Teeth (I)	0.2
Bottom Radius (UR)	1.5

**Table 2:** Variation range of the data and simulation step size.

Variation Parameter	Simulation Range	Simulation Step	Base Value
t (s)	0∼20	0.1	0
T (K)	−233.15∼343.15	5	293.15

## Data Availability

The data presented in this study are available in the article.

## Abbreviation

The following abbreviation is used in this manuscript:

M-μHRG	Metal-μhemispherical resonant gyros
